# Hospital and Institutional News

**Published:** 1915-10-30

**Authors:** 


### HOSPITAL AND INSTITUTIONAL NEWS.

### HIS MAJESTY'S GIFT TO THE CANADIAN RED CROSS.

It is announced that the King, on hearing that the Medical Service of the Canadian forces was looking for a house near London to serve as a convalescent home, commanded the Lord Chamberlain to offer Upper Lodge, on the Royal estate at Bushey Park. This gracious offer has been accepted on behalf of the Canadian forces by Surgeon-General Coleton Jones, director of their Medical Services; and the new home will be furnished at once under the superintendence of Colonel Hodgetts, Chief Commissioner in England of the Canadian Red Cross. The staff will be composed entirely of members of the Canadian Army Medical Corps. Everyone knows the warm interest of His Majesty in everything that appertains to the care of the sick and wounded, as well as to the affairs of his Oversea Dominions; and this timely loan is merely an additional evidence of both those sympathies. His Majesty's personal knowledge and appreciation of hospital requirements and hospital problems are sufficient guarantee that the buildings thus generously placed at the disposal of the Canadian Medical Service are suitable for the purposes they are to be put to: when spring and summer come again we can imagine few more ideal places than Bushey Park for the restoration to health of the Empire's shattered heroes.

### A BIG STEP FORWARD.

It is not often that a Parliamentary paper contains a proposal so far in advance of current thought as does the Report for 1914-15 of the Medical Officer to the Local Government Board, issued on October 20. The idea in question is one of a series of measures suggested as possible aids to the reduction of the mortality of women in childbirth, and is nothing less than the notification of pregnancy. The preventible mortality of childbirth has often been the subject of comment in these columns, but we do not remember ever to have seen before so bold and drastic a remedy for an evil the suppression of which is certainly worthy of every feasible effort. It is pointed out in the Report that there is in Scotland one maternal death to every 175 registered births; in Wales, one to 179; in Ireland, one to 191; and in England, one to 256; and that the majority of these deaths are due to preventible causes. The superiority of the English statistics would seem to point to the benefits of a Midwives Act for Scotland and Ireland. Among other remedies suggested for these much too high mortality figures are fuller inspection of midwives, ante-natal clinics and visiting, provision of skilled assistance at maternity centres, and other expedients. But the chief interest of the Report is certainly contained in the far-reaching proposal for the notification of pregnancy. Whether or not it is too far ahead of public opinion to be practicable now, it is probably destined to arrive some day.

### OPHTHALMIC SURGERY BY NURSES!

Either a cock-and-bull story has been foistered by some practical joker on a youthful reporter, or else things are not as they should be at "a London hospital." The story in itself contains such elements of improbability that we prefer to accept the former of these alternatives. It is stated, with the utmost gravity, by the *Daily Chronicle* that "a professional man who had found it impossible to get an oculist to attend to his eye called at a hospital, hoping that in return for a donation to the funds one of the doctors would be able to attend to him." It is, of course, quite inconceivable that in London anyone seeking an ophthalmic surgeon for a non-urgent complaint should have the slightest difficulty in finding one, and the action taken was, equally plainly, a gross attempt at abuse of a voluntary hospital. The story proceeds to the effect that preferential treatment before a crowd of waiting out-patients was refused, but a "sister" volunteered to perform the requisite operation. The "professional man" consented; "the eye was flooded with cocaine by the competent sister, and in the gentlest and most skilful way she removed a troublesome cyst." Furthermore, the credulous reporter is informed, it is not at all uncommon "for the matron or senior nurses to undertake these minor operations." We devoutly trust that this preposterous story has no foundation, and that there is no hospital in London or elsewhere in which the diagnosis and operative treatment of diseases of the eye or any other part are entrusted to unqualified persons, whether they be matrons, nurses, or lift-boys; and we shall require more than assertions in the lay press to induce us to believe that this is the case.

### KING EDWARD VII.'S HOSPITAL CARDIFF.

At the monthly meeting of the board of management of this hospital a fine compliment was paid to Colonel Bruce Vaughan, when it was unanimously decided to call the new wing of the hospital the "Bruce Vaughan Wing." The following were the terms of the resolution, which was gracefully proposed by Major-General Lee, supported by every section of the widely representative committee, and carried with acclamation: "That, as an appropriate recognition of Colonel Bruce Vaughan's most valuable services to the hospital, the additional buildings at present known as the 'New Wing,' and which have been erected as the result of his foresight and unselfish devotion, be known for the future as the 'Bruce Vaughan Wing.'" It is well known in the hospital world that Colonel Bruce Vaughan's energy and personality have made the Cardiff hospital what it is, so that the recognition is indeed "appropriate." At the same time it should prove an encouragement to hospital workers generally—who are, after all, only human—to know that their unselfish devotion is appreciated. The Bruce Vaughan Wing is a magnificent pile of buildings, accommodating nearly

100 beds and comprising also many modern hospital adjuncts. Colonel Bruce Vaughan himself has been the honorary architect of the building, which cost, with its equipment, between £40,000 and £50,000.

### POOR-LAW AMALGAMATION IN GLASGOW.

The most recent proposal for the amalgamation of Poor-Law Unions comes from Glasgow, and originates from the Glasgow Parish Council. The present suggestion is to combine the Poor-Law areas of Glasgow and Govan. The original idea was to create a new Poor-Law authority, the area of which should coincide with the municipal boundaries of the city. To effect this would have required, however, an Act of Parliament. The Glasgow Parish Council has therefore decided for the present upon the smaller plan, which needs only the sanction of the Secretary for Scotland. The agitation for the union of the Glasgow and Govan parishes has been going on for some time. It has been brought to a head by a scandal which has caused much local indignation. A woman applied to the Glasgow parish authorities for admission to their Poor-Law hospital for her confinement. She was referred to Govan. Govan referred her back again to Glasgow, where admission was again refused. In her despair she threw herself into the Clyde, from which she was rescued, and her baby was born on the ferry steps to die a few hours later. A union of the parishes would lead not only to an equalisation of the poor rate and the placing of its burden upon those best able to bear it, but would also make for economy by preventing the duplication of institutions needed for indoor relief. The importance of the proposal may be gauged by a consideration of the populations involved—577,559 in the case of Glasgow, and 363,571 in the case of Govan.

### RANCID BUTTER AT BERMONDSEY.

Some four months ago the medical officer of health for Bermondsey and the Town Council of that borough attempted unsuccessfully to lay an embargo under the Unsound Food Regulations upon eighty-two casks of imported butter. The full circumstances of this attempt, and some comments upon the principles at stake, are contained in a circular issued by the medical officer of health, and are well worthy of the attention both of sanitary and of medico-legal authorities. The butter was rancid, and was shown by analysis to contain 3.16 per cent. of free fatty acids; the normal content is well under 0.5 per cent. It was argued on these premises that the butter was unfit for human consumption. For the defence, it was urged that while the butter was not fit to be sold over the counter, and was not intended for such sale, yet it was fit for use in making cakes or confectionery, where its rancidity would be disguised by other flavourings. The Court seems to have acted on this view, and upon the lack of proof for the prosecution that any definite human ill must ensue upon the consumption of this butter.

### THE M.O.H.'S SARCASMS.

That such a view is short-sighted, even erroneous, is easily arguable, and it provides the medical officer of health with a target for some effective shafts of sarcasm. He declares that the War Office, Boards of Guardians, and similar authorities would not accept butter such as this, and that only the general public fails to secure protection against foodstuffs made of impure or unsound materials whose rottenness is concealed by other flavouring. He pleads for more effective control of the confectionery and some similar trades in which this kind of semi-adulteration is rife. That the law should exempt from the provisions of the Unsound Food Laws foodstuffs of poor quality compounded of unsound materials is, as the medical officer of health very justly suggests, a serious matter, and it is not surprising to note that the firm which bought this butter did not wish to have its name disclosed. Further, the defence invoked the evidence of some well-known analysts, whose pretensions as experts in these matters Dr. King Brown derides; on grounds of public utility he makes some strictures upon them which seem to be not devoid of foundation. The purchasing firm's name, however, should be published widely in association with this transaction.

### SHOULD THE BIRMINGHAM ORTHOPÆDIC HOSPITAL BE REBUILT?

The position of the special hospitals in war time is one of some interest, and the position of affairs at one of the few orthopædic hospitals in the country deserves a brief record. The only hospital in the Midlands devoted to the treatment of deformities is the Birmingham Royal Orthopædic and Spinal Hospital. This institution has, of course, to provide treatment for many cases who require a prolonged stay in the wards, and at the same time is hoping to embark on an extension and rebuilding scheme. It is true that, since the outbreak of war, the number of patients has somewhat fallen off, but the committee feel that with a suitable modern building containing up-to-date wards much more work could be done which at present is left unattempted. Looking ahead, moreover, the committee believe that many cases of injury to soldiers will have to be referred to the hospital, and would therefore like to be ready to deal with them in such a way as to consolidate and extend the reputation of the hospital in the special phase of treatment to which it is devoted. What is really hoped for is rebuilding, but whether the hospital can make out a case sufficiently convincing to secure the necessary funds appears doubtful.

### THE PLANNING OF OPEN-AIR HOSPITALS.

When Colonel Griffiths, the officer commanding the 1st Eastern General Hospital, Cambridge, paid a visit of inspection to the new Huddersfield War Hospital (which was lately described in The Hospital) he pointed out certain improvements which the latter institution contained. Modelled though it was on the open-air plan adopted at Cambridge, Colonel Griffiths remarked on the additional comforts which the voluntary funds at their disposal had enabled them to embody in the Huddersfield plans. Instances of these he found in the separate dressing-rooms to which patients can be taken to have their wounds attended to, and where the anæsthetics sometimes necessary during the process can be conveniently administered. These rooms, which he had embodied in the Cambridge plans, were struck out, but he was very glad to note their inclusion at Huddersfield. Another point on which he congratulated them was the large administrative block. He remarked on the need for ample office room for the registrar and the matron. Dealing with structural points, Colonel Griffiths insisted on the need for providing the nurses with the best accommodation, which in the war hospitals was not always easy, and also emphasised the value of noiseless blinds, and was sufficiently pleased with those at Huddersfield to ask for all the practical details concerning them. Lieut.-Colonel G. S. Woodhead, Professor of Bacteriology at Cambridge University, is a Huddersfield man, and he, too, has given valuable assistance in placing at the service of the committee the experience gained at Cambridge.

### RED CROSS DAY IN THE STREETS.

Anyone who was compelled to travel during the past week so as to be in London on Red Cross Day, in the provinces on the next, will have noticed a difference in the manners of the street collectors. In London if anyone purchased a Red Cross he was tactfully let alone by the other collectors, in accordance with the plan originally agreed on that the purchase of some flower or sign should leave the purchaser free from further solicitation. In the provinces, however, this assumption was not acted upon, and we noticed with amusement many persons decorated with flags who were still asked to purchase further supplies by the street collectors. Indeed, the more flags people wore the more they were asked to buy—a practice which in the long run is not likely to make street collections more popular.

### FIFTY MOTOR AMBULANCES SUNK.

It is announced by the French Relief Fund that the fifty motor ambulances which were inspected by Her Majesty Queen Alexandra, and afterwards handed over to the French Government, have been entirely lost. The ambulances were handed over in London to a representative of the French Government for shipment to France. As the French Government took all risk in them, the Relief Fund did not insure the cars; and it is now learnt that they are a total loss owing to the ship on which they were dispatched having been torpedoed by a German submarine. The committee of the French Relief Fund are most desirous of replacing this fleet of ambulances as quickly as possible, and are appealing to all sympathisers with France for donations for this purpose; the honorary secretary will be pleased to receive them at 83 Pall Mall, S.W. It is especially to be regretted that this particular cargo should have been lost, since it represented in such concrete form the sentiments of the people of England for that of France. The fact that the gift had already been handed over sufficiently attests the spirit of the sympathy between the two countries.

### SUSPENSION OF SCHOOL MEDICAL INSPECTION.

A bold step has been taken by the Aberdeen County Committee on Secondary Education in suspending entirely within their district the medical inspection of school children, in order that their assistant medical officer may join the R.A.M.C. The Medical Inspection Sub-Committee were in favour of allowing the senior medical officer to carry on the work as far as he could; but an amendment was moved and carried that his services too should be dispensed with, so as to set him free for war work. We are not insensible of the benefits of school medical inspection, nor of the serious disadvantages of interrupting it once it has been begun; but we do feel that the Aberdeen Committee has struck the right note, and that in times of real stress such as now prevail the whole organisation of school inspection should be overhauled so as to free as many medical men as possible. As a speaker at Aberdeen remarked, we managed to get along for many years without school doctors, and can do so again until the end of the war.

### THE PROSPECTS OF ANOTHER SPOTTED FEVER OUTBREAK.

An important discussion on this disease took place at the Royal Society of Medicine on October 19. Sir William Osler, who opened the debate, dwelt on the probability of a further outbreak this winter, especially among the military; and confessed his faith in Flexner's serum treatment, in spite of the many failures which have been reported. Surgeon-General H. D. Rolleston summarised the experience of the Royal Navy since the war began, from which it appears that simple lumbar puncture gave much better results than any variety of serum treatment; the latter was also easily surpassed by the results of soamin injections. Dr. Robb, of Belfast, on the other hand, has had very favourable results from the use of Flexner's serum, and believes in it. The debate was concluded by two bearers of historic names in physiology, Captain Michael Foster and Captain J. F. Gaskell; both these officers have had much better results from simple lumbar puncture than from serum therapy. Sir W. Osler's prognostications are somewhat alarming, but we must hope they will not be fully realised: at any rate, the health authorities are forewarned.

### POOR-LAW RELIEF FOR DISABLED SOLDIERS.

The Guardians of the East Preston Union received at their last meeting an application for the admission to their infirmary of a soldier paralysed as a result of a wound inflicted during the battle of the Marne. The man has a pension of 25s. a week, but his friends find it impossible to give him the necessary nursing attention. The Guardians decided to admit the man and to charge his pension with the cost of his maintenance. Cases such as this are likely to prove numerous, and it is incumbent upon the nation to see that proper provision is made for them. Poor-Law infirmaries are admirably adapted to deal with such cases, and the one argument against their use for this purpose is the stigma which in public opinion still attaches to their inmates. That stigma can only be removed by a reform of the whole Poor-Law system such as was suggested in the Minority Report of the Royal Commission on the Poor Laws. All Poor-Law infirmaries of the best type after the war should be reorganised and made into State hospitals, as we have insisted continuously for years.

### THE LONDON AMBULANCE SERVICE.

Having been for many years persistent advocates of an ambulance service for London, we note with satisfaction that the scheme devised last year by the London County Council has at length been brought into operation. Six ambulance stations have been opened, three on each side of the Thames. Five of these are equipped with one motor ambulance each, and one station has two; in addition, the motor ambulance at the Hampstead General Hospital is available for use in connection with the scheme. The service is for street accident and street illness cases, and for cases of accident in factories, private houses, etc.; but not for the conveyance of sick persons from private houses. The cost has been about £7,500, and the annual upkeep is estimated at £10,000.

### THE RED CROSS AT BURLINGTON HOUSE.

The President and Council of the Royal Academy have handed over to the British Red Cross Society one wing of their premises at Burlington House for use as central workrooms during the winter months. The accommodation thus secured is to be used as an administrative and organising centre for all Red Cross working parties throughout the country. Arrangements have been made for the workers to be included under the Government scheme for the recognition of voluntary effort; all who perform three months' satisfactory work under the Red Cross are to receive the Government badge. The working hours will be from 10 a.m. to 1 p.m., and 2 p.m. to 5 p.m., Saturdays and Sundays excepted. Working overalls will be provided, but must be kept clean at the member's expense. The subscription (for those working at Burlington House) has been fixed at five shillings. It is hoped that all working parties throughout the country will register at Burlington House.

### DEATH OF A HOSPITAL COMMANDANT.

It is announced that Mr. Charles Thomas Bruce died on October 23, at his residence in London, of enteric fever, contracted while on duty as commandant of a hospital unit in Flanders. There have been few deaths during the campaign hitherto from this until lately dreaded scourge of armies, and it is particularly unfortunate that one should be carried off who was devoting his services to the care of the sick and wounded. It is evident, of course, that Mr. Bruce, being a civilian, was commanding some form of voluntary organisation, not a proper Army hospital; and it may be that camp sanitation was less carefully supervised than in the much-inspected regular hospitals, where enteric fever amongst the staff is practically unknown. Mr. Bruce was a grandson of the seventh Earl of Elgin, and a member of the Royal Yacht Squadron: he had been twice married, the second time only a year ago.

### THE FORFARSHIRE LOCAL AUTHORITIES AND SANATORIUM TREATMENT.

The question of the payment for the treatment of insured persons in the Noranside Sanatorium is still agitating the Forfar District Committee, and at a recent meeting further stimulus was given by a letter from the Local Government Board. In this communication it was pointed out that, in view of the decision of the Forfar County Council not to adopt the section of the Insurance Act relating to the administration of the sanatorium treatment by a central body, it would be the duty of all local authorities in the county to submit schemes dealing with their respective districts. Until such schemes were approved the Board would not be able to distribute any portion of the capital grant in respect of Noranside Sanatorium. It is understood that this grant amounts to a sum of £4,000. The Chairman of the meeting, Mr. D. M. Graham, among other objections, cited the one that all the Parish Council representatives would be swept out of doors if they had only one area. To an outsider this does not appear a terribly alarming prospect, and it will probably happen in the end that a scheme for the larger area will have to be adopted. Meanwhile the formulating of a suitable reply to the Local Government Board was entrusted to three officials, including Dr. Sinclair, the county medical officer.

### THE ARCHBISHOP AT THE CROYDON GENERAL HOSPITAL.

We are glad to welcome back to health and work the Archbishop of Canterbury, whom we cordially congratulate on his recovery. The Archbishop, with Mrs. Davidson, paid a surprise visit on Saturday afternoon to the above hospital, of which his Grace is president. The principal wards, including those occupied by wounded and sick sailors and soldiers, were visited, and the wounded were cheered by the interest displayed in each of them by the visitors. His Grace was accompanied by the honorary chaplain, the Rev. Canon White-Thomson, Vicar of Croydon and Rural Dean. We are glad to note the fact that the Archbishop of Canterbury is following the example of Their Majesties the King and Queen in making surprise visits to our hospitals, and rejoice to know that he was much pleased with his visit and interested in the excellent work which it was his privilege to inspect. As we have said on a previous occasion, there are few hospitals which better repay a prolonged visit than the Croydon General Hospital, which has many merits, not the least of them being the possession of most capable officers, and the maintenance of its administration at the highest point of efficiency.

### A SPONTANEOUS AND INSPIRING GIFT.

A Dutch gentleman, Mr. N. W. Burlage, of Utrecht, Holland, being in London in July, was moved by the sight of the wounded soldiers sitting outside Westminster Hospital. On his return to Holland he decided to apply his sympathy in personal service, feeling so sorry for the wounded, and set to work to interest his fellow-countrymen in our warriors. At present Westminster Hospital has eighty-one sick and wounded soldiers amongst its patients, and on the 15th instant Mr. Burlage called on the secretary there and paid to him a sum of £132 1s. 2d. which he had collected in Utrecht, The Hague, Amsterdam, Rotterdam, and other places in Holland. The subscribers to this fund numbered seventy-one, of whom he left a list, the individual subscriptions varying from 1s. to £50. The subscribers' object in giving their money is expressed by them to be "to show their sympathy with the British wounded soldiers in the Westminster Hospital of London, and the undersigned Hollanders hope that the sum, although small, will be accepted in the same good spirit as it is offered." This incident has an international interest and importance as great as it is gratifying and honourable to all concerned. It demonstrates a new purpose which the hospital verandahs may often fulfil with advantage to a voluntary hospital, while they have immeasurably added to the hygienic efficiency and curable qualities of our great palaces of pain.

### THE LATE DR. W. G. GRACE.

It is not as a medical man that "the Doctor" was known to millions of the public, though he practised medicine for many years in Gloucestershire. In fact, it was fourteen years before he qualified as a medical man that William Gilbert Grace attained fame such as few of the most eminent surgeons or physicians can aspire to. Certainly he had qualities of hand and eye which might have made him a great operator; and in physique he excelled even the giants of the pre-anæsthetic period, when a great surgeon was said to require the strength of a blacksmith as well as the nerves of a lion-tamer and the hands of a violinist. But cricket was, naturally, his greatest as it was his earliest preoccupation; though medicine seems to have been a good second. How great "the Doctor" was not even "averages" can really explain: for years and years he was beyond all rivalry or thought of it, acknowledged prince of the art of cricket as no single man has ever been hitherto. As for his matchless staying powers, the story of his career when his prime was past teaches us that even at strenuous athletic pursuits a man who will take care of himself physically can continue to take a prominent part long after most men think it necessary to let themselves run down.

### HOSPITAL LAUNCHES FOR THE DARDANELLES.

Some interesting particulars have been given in the *Liverpool Journal of Commerce* about a new type of hospital ship for use at the Dardanelles. Six of these "hospital launches" are on order, and the first of them has already left the stocks. They are forty-two feet in length by nine feet beam; they have a flush deck for three-quarters of their length forward, carried on built-up topsides. The ward is arranged amidships, with accommodation for twelve stretcher cases; forward of this is the engine space for a two-cylinder petrol engine driving a solid propeller, with a reverse gear. The remaining forward portion of the vessel is given over to a forecastle, with living accommodation for two of a crew. The vessel is steered and controlled from the weather deck and can be handled by one person; that is to say, the engine controls can be reached and manipulated by the man steering. Official trials have been carried out, and it is said that a very satisfactory speed has been attained.

### EXPANSION AT TOXTETH INFIRMARY.

The Toxteth Board of Guardians has decided to offer the War Office an additional hundred beds in the new workhouse infirmary in case of emergency. This decision was not arrived at without some debate, in the course of which two arguments were brought forward against the proposal. One was that so long as the various strictly war hospitals are not full, there is no need for any further offers of help to be made to the War Office: this argument is not very impressing, since the Army authorities must obviously be allowed to decide whether more accommodation is (or may be) wanted. The other objection seems to be better founded—namely, that it is inexpedient to house paupers and soldiers in the same buildings. With this view we have considerable sympathy, at least so long as there is sufficient accommodation elsewhere. It appears from the report of the discussion at Toxteth that soldiers are already received in the infirmary; but we cannot say we approve of this admixture of patients in the same building.

### INCREASED PRICES AT LEICESTER INFIRMARY.

The chief problem of the work of the Leicester Royal Infirmary, submitted to the quarterly meeting of the governors, was the increase in the cost of maintenance. The only decrease in provisions was one of £5 in malt liquors—from £19 to £14. The larger number of patients, military and civil, did not alone account for the increased expenditure, the enhanced prices amounting in some of the items to 25 per cent. and upwards. Fortunately, the income showed no diminution, but in times of financial uncertainty like the present the need of an expanding income was sharply accentuated. Legacies had too often been relied on for times of difficulty, but it was hoped that in the present undoubted prosperity of Leicester's industries an augmentation of the institution's finances would be found in generous donations. In moving the adoption of the report, Colonel C. J. Bond alluded to the administrative difficulties in maintaining the increasing work, and to his own work in the operative treatment of physical disabilities in recruits. Of the three hundred so treated, a large proportion reported themselves as having joined the Services. One had received a commission, others had been wounded in action, and many were in training.

The problem of the unfit citizen was a national one which he felt convinced should not be left for great emergencies like the present to disclose or remedy.

### THE PAY OF THE "LOCUM TENENS."

The current scales of remuneration for a *locum tenens* in a medical practice were rising steadily before the war, and had been doing so for some years. Since that time the rates have jumped by leaps and bounds, and are now at least twice as high (more rather than less) as they had ever been previously. This arises, clearly, from the concurrent diminution in the supply and increase in the demand created by the war, and as an economic phenomenon is normal enough. It is not easy to see, therefore, that any marked decline in prices is likely to happen, short of interference from outside (by the State). The *Queen*, commenting on the matter, roundly denounces the lack of patriotism of those who accept these inflated stipends; but is unable to suggest any remedy now applicable. The editor of that journal admits that women would not be well received in general practice as substitutes for men; but holds that they should in times past have received more encouragement to enter the medical profession. If more women had qualified, it is argued, not only would more men be able to join the R.A.M.C., but also the public prejudice against women doctors would have vanished. The sequence of the argument depends, it will be noticed, on various might-have-beens, the evidence of which is the *ipse dixit* of the editor. We admit that the *locum tenens* is overpaid just now, but we do not see any feasible alternative; and at least for generations past he has been underpaid.

### THE WAR AND PUBLIC HEALTH.

Dr. G. B. Millson, medical officer of health for the Borough of Southwark, in his annual report refers to the disadvantages under which public health authorities are at present labouring. "It has only been with the greatest difficulty that the district has been kept in a fairly good sanitary condition. Much more work was thrown upon the sanitary staff owing to the scarcity of labourers, especially of skilled workers. We have had many changes in the staff owing to so many of the men enlisting. Up to the present the work of the department has been efficiently carried out." Housewives have been urged to burn as much rubbish as they can, but a more strenuous appeal is urgently called for. Public health authorities have to deal with immense quantities of trade refuse which they are called upon to cart away. Traders and manufacturers might be forced to burn as much as they can of this refuse at their works. The call for sterner methods is shown by a recent incident at the Elephant and Castle, in South London. Three street merchants had done a roaring trade in the sale of bananas, taking on the Saturday over £18. They piled the rubbish from their boxes in a great heap on a piece of waste land close by. Needless to say, when it was discovered they were called upon at once to remove it.

### THE WOLLASTON WAR HOSPITAL OPENED.

On Monday last the Newport section of the 3rd Western General Hospital was opened. Known locally as the Wollaston War Hospital, it has accommodation for 714 patients, and the chief officer is Major Wilson. Wollaston House had originally 600 Poor-Law patients, who have now been distributed among other institutions. A good deal of local interest has been excited, since the new institution is the largest self-contained section in the Western Hospital district.

### DAMAGES AGAINST AN "INSTITUTE."

A much advertised "Institute" carrying on a large unqualified practice by means of electrical methods has been cast in damages to the extent of £48 odd by a jury at the suit of a lady in Maida Vale. What appears to have occurred is that the plaintiff was burned in three places on the knee while undergoing "diathermy" treatment; and it was alleged on her behalf, though denied by the defence, that the proprietor of the "Institute" was so constantly nagging and scolding the "nurse" who applied the treatment, that the latter became nervous and made mistakes. The moral of the whole affair is, in our opinion, that unqualified practitioners, whether as managers or as proprietors of self-styled Institutes, are not fit persons to diagnose disease and treat it by remedies so potent as those in the armamentarium of electro-therapeutics. As, however, the law does not forbid these practices, it is at least some advantage to learn that, in spite of quibbles as to proprietorship, there is some legal remedy for negligence and *malapraxis*.

### THIS WEEK'S DRUG MARKET.

The advance in the price of quinine has continued, and a large business has been done at rates which a month ago few people would have thought possible. To some extent the great advance is no doubt due to speculation, but other influences have been at work, not the least important of which is the considerable buying on the part of America. Whether prices will continue to tend upwards it is difficult to say, but there is certainly no indication at present that prices are likely to decline. The high value of opium is well maintained, but there has not been any advance in makers' quotations for morphine and codeine. Bromides are again dearer. Higher prices have been paid for ipecacuanha. Business is being done more freely in cod-liver oil at high prices, and there is little reason to think that there will be any substantial decline during the winter; in fact it is by no means improbable that with the near approach of the chief consuming season, the tendency of values will be in an upward direction. In synthetic drugs there have been no particularly noteworthy advances in prices, but several of the most important of these drugs are only obtainable in small quantities. Peppermint oil and menthol tend upwards in value. No further change has been made in the quotations for glycerine, but it is not improbable that higher prices will rule shortly.

